# Comparison of cataract patients with regular corneal astigmatism after implantation of extended range-of-vision and bifocal toric intraocular lenses

**DOI:** 10.3389/fmed.2023.1105876

**Published:** 2023-10-02

**Authors:** Zhuoya Li, Rong Guo, Xiaomin Hu, Xinyue Yang, Ziyuan Wen, Yi Lin, Hui Zhang

**Affiliations:** Department of Ophthalmology, The Second Hospital of Jilin University, Changchun, Jilin, China

**Keywords:** refractive cataract surgery, astigmatism, extended range-of-vision IOLs, high-order aberration, visual quality

## Abstract

**Purpose:**

To compare the postoperative visual acuity and visual quality between extended range-of-vision and multifocal toric intraocular lens (IOLs) after implantation in cataract patients with regular corneal astigmatism.

**Setting:**

Department of Ophthalmology, the Second Hospital of Jilin University, Changchun, Jilin Province, China.

**Design:**

Retrospective and single-center study.

**Methods:**

The study involved implanting the Tecnis Symphony (ZXR00IOL) or the bifocal toric (ZMTIOL) in patients undergoing cataract surgery. Three months after surgery, lens performance was evaluated using distance, intermediate, and near visual acuity tests, defocus curves, the modulation transfer function (MTF), a visual function index questionnaire (VF-14), and the adverse optical interference phenomena.

**Results:**

The 3-month postoperative follow-up found that both groups had good corrected distance vision. The ZMT group had better-uncorrected distance visual acuity and near visual acuity (*p* < 0.05). However, the ZXR group showed better uncorrected intermediate visual acuity (*p* < 0.05) and visual continuity. Overall astigmatism in the postoperative ZMT group was significantly lower than that in the pre-operative group (*p* < 0.05). The ZMT group had lower total high-order aberrations (tHOs), higher MTF values, and higher VF-14 scores (*p* < 0.05). Finally, the ZXR group exhibited reduced halo and glare phenomena (*p* < 0.05).

**Conclusion:**

We found that ZMT can effectively correct a corneal astigmatism of 1.0–1.5 D and ZXR can improve patient outcomes regarding subjective optical quality and range of vision. These findings have the potential to improve future astigmatism treatment options.

## 1. Introduction

Cataract surgery has entered the era of refractive surgery. Multifocal intraocular lens (MIOLs) can replace the opaque lens of cataract patients and solve the problem of ametropia (1). Among these lens, the diffractive IOL uses a diffraction ring to split incident light into 2–3 focal points. Furthermore, the continuous-range diffracted IOL provides a power of 1.75 diopters (D), which causes ladder diffraction to allow for extended vision. However, while multifocal IOL technology offers high visual acuity, it can also produce adverse optical interference phenomena, such as glare and halos (2). Another limitation is that they cannot correct corneal astigmatism for patients, a common type of ametropia. Approximately 40 and 20% of cataract patients exhibit astigmatism greater than 1.0 D and 1.5 D, respectively, prior to surgery (3). Studies have established that pre-operative astigmatism above 1.0 D can significantly impact the patient’s postoperative visual acuity, contrast sensitivity, and quality of life (4, 5). Thus, it is crucial to address pre-operative astigmatism when using multifocal IOLs to correct farsightedness and myopia.

The toric IOL has been in clinical use since 1992. A meta-analysis study by Kaur et al. (6) indicated that, for patients with pre-operative astigmatism, the toric IOL offered improved uncorrected distance vision, a higher spectacles independence, and lower residual astigmatism compared to the non-toric IOL. The complex surface design of Tecnis ZMT (Abbott Medical Optics, United States) diffraction bifocal toric IOL is used to correct hyperopia, myopia, and astigmatism. Although the bifocal IOL distributes light to two points, some light energy loss occurs, resulting in glare and halo phenomena (7). However, the Tecnis Symphony (ZXR00, Johnson & Johnson, United States) extended depth of focus (EDoF) IOLs extend the depth of focus and increase the tolerance of residual astigmatism, due to their unique diffraction grating design (8, 9). Unfortunately, there is a lack of studies on the effects of toric bifocal IOLs on postoperative visual quality (10, 11). Previous research has described several aspects of visual outcomes, including visual acuity, defocus curve, contrast sensitivity, rotation, subjective optical phenomenon, and use of spectacles. However, to our knowledge, research involving objective visual quality measurement has not yet been published, which is a crucial factor in assessing the patient’s visual outcome after IOL implantation. Thus, the objective of this study is to provide further insight into this vital subject matter. In this study, the visual quality of EDoF IOL ZXR00 and toric bifocal IOL ZMT in patients with pre-operative astigmatism between 1.0 D ~ 1.5 D were compared and analyzed. Through the comparison of the postoperative visual acuity, visual quality, spectacles independence, and questionnaire results of the two groups, our aim is to offer essential information to guide refractive cataract surgery for clinicians.

## 2. Methods

### 2.1. Research objective

This retrospective study was approved by the institutional review board of The Second Hospital in Jilin University, Changchun, China and underwent ethical review at our hospital. The ethics review number is 2022–229. The study was performed in accordance with the tenets of the Declaration of Helsinki. The patients provided written informed consent to participate in this study.

### 2.2. Inclusion and exclusion criteria, grouping, and pre-operative examination

Patients underwent uneventful cataract surgery with the implantation of a Tecnis ZMT (Abbott Medical Optics, United States) or a Tecnis Symphony (ZXR00, Johnson & Johnson, United States) IOL. The surgeries took place from January 2021 to July 2022 at our hospital.

The inclusion criteria were as follows: (1) pre-operative diagnosis of cataract and age > 50 years; (2) regular corneal astigmatism in the range of 1.0–1.5 D; (3) angle of kappa and alpha <0.5; and (4) photopic pupil >2.0 mm and mesopic pupil <6.0 mm. The exclusion criteria were as follows: (1) history of ophthalmic surgery, trauma, uveitis, retinopathy, glaucoma, high myopia, or severe dry eyes; (2) irregular corneal astigmatism; (3) intraoperative complications; and (4) severe diabetes, immune diseases, and systemic diseases.

All patients underwent the following examinations before operation: uncorrected distance visual acuity (UDVA, 5m), best-corrected distance visual acuity (CDVA, 5 m), intraocular pressure (IOP), tear secretion, biological measurement (IOL Master 700, Carl Zeiss Meditec AG), corneal topography (OPD-ScanIII, NIDEK), slit lamp examination, binocular ultrasound, optical coherence tomography (SPECTRALIS OCT, HEIDELBERG), and corneal endothelial count and morphology.

### 2.3. Calculation of IOLs and labeling method for toric IOLs

Refractive parameters were measured using an IOL Master 700 (Zeiss, Germany). IOL power was calculated using the Barrett TK Universal II formula, and the target refractive diopter was 0 ± 0.5 D.

An online calculation platform^1^ was used to calculate the ZMT models and determine the position of the operative incision and IOLs loop axis. Before surgery, we marked the axial and operative incision positions on the patients.

### 2.4. Operation method

The same surgeon operated on all patients. Before each operation, the operative eyes were fully anesthetized using 0.4 ml:2 mg procaine hydrochloride. A 2.2 mm main corneal incision, 0.8 mm side-port corneal incision, and 5.5 mm diameter circular continuous capsulorhexis were performed. Lens extraction was accomplished using a standard phacoemulsification technique. The IOL was implanted into the capsule bag, and the toric IOLs were rotated to align with the axial position of the pre-operative marker. Both the toric and EDoF IOLs were centered. No complications occurred during the operations.

### 2.5. Intraocular lenses

The EDoF TECNIS ZXR00 has a one-piece posterior surface diffractive design with an EDoF IOL. It has nine grating diffraction apertures on the rear surface, and the Echelette diffraction grating technology achieves a continuous field of view; chromatic achromatic technology is used to further enhance the image contrast (9, 12, 13). A large central optic design with a diameter of 1.6 mm increases tolerance, has a strong anti-deviation ability, and can accommodate astigmatism <1.5 D.

ZMT IOL integrates aspheric, diffractive multifocal, and toric designs, and has an all-optical rear surface diffraction design, with +4.0 D attached to the near side. ZMT IOL is a pupil-independent IOL with the same ratio of far and near focus under photopic or mesopic photometry. It can correct different degrees of astigmatism of the cornea according to the different cylinders (9).

### 2.6. Postoperative visual quality assessment

#### 2.6.1. Visual acuity

Three months after the operation, a standard logarithmic visual acuity chart was used to measure uncorrected distant, intermediate and near visual acuity (UDVA, UIVA, and UNVA) at 5 m, 80 cm, and 40 cm, and corrected distant visual acuity (CDVA) at 5 m. All patients were assessed in an environment of equal luminance.

#### 2.6.2. Defocus curve

The defocus curve was drawn using a comprehensive optometer and performed with uncorrected visual acuity. The optometer adjusted the degree of the spherical lens in front of the operated eye. The defocus curve ranged from +2.0 D to −4.0 D (by decreasing the spherical degree by +0.5 D for each reading).

#### 2.6.3. High-order aberration and MTF

Total high-order aberrations (tHOs) [including spherical aberrations (SA), coma, and trefoil aberrations] and the MTF values were measured at a pupil diameter of 3 mm using an iTrace visual quality analyzer (Tracy Technologies, United States).

#### 2.6.4. Spectacles independence, questionnaire, and subjective adverse optical interference phenomenon

A visual function index questionnaire (VF-14) was used to evaluate visual function in patients (14). There were 14 items, all divided into five grades according to their degree of difficulty. Adverse optical interference (glare and halo) and the spectacle independence of the postoperative patients were also evaluated.

#### 2.6.5. Refractive state

The iTrace visual quality analyzer was used to measure (i) the pre-operative and postoperative corneal astigmatism (*D*) and the whole total astigmatism (D); (ii) the postoperative residual astigmatism (*D*) of the two groups; and (iii) the axial deviation (*D*) of the ZMT IOL with the toric check function.

### 2.7. Statistical analysis

SPSS25 (IBM, Armonk, NY, United States) was used for the statistical analysis. First, the Kolmogorov–Smirnov test was used to test for a normal distribution of data. When a normal distribution was found, two independent samples Student’s t-test was used; the results are expressed as mean ± standard deviation. If the data did not follow a normal distribution, a nonparametric rank-sum (Wilcoxon) test was used to test the difference between two independent samples. The ratio of the two groups was compared using Fisher’s chi-square test. All tests were double-tailed statistics, and statistical significance was set at a *p-*value of <0.05.

## 3. Results

### 3.1. Pre-operative parameters

Based on the inclusion and exclusion criteria, 95 patients (103 eyes) were included. ZXR00 IOL was implanted in those who required intermediate vision and ZMT IOL in those who required near vision. There were no significant differences in age, eye difference, sex, corneal astigmatism, axial length, intraocular pressure, etc., between the two groups (*p* > 0.05) (Table 1).

**Table 1:** Comparison of general data between the two groups before surgery.

Preoperative parameter	Mean ± SD		*p-*value
	ZXR	ZMT	
No. of eyes (patients)	53 (46)	50 (49)	
Age (*y*)	58.77 ± 11.29	61.30 ± 7.15	0.310
Sex (*n*)			0.738
Male	29	29	
Female	24	21	
Eyes (*n*)			0.896
OD, ocular sinister	29	28	
OS, ocular sinister	24	22	
Astigmatic (*D*)	−1.27 ± 0.14	−1.29 ± 0.14	0.380
Anterior chamber depth (mm)	2.99 ± 0.41	3.02 ± 0.50	0.709
Axial length (mm)	23.13 ± 1.38	23.09 ± 1.43	0.140
IOL power (*D*)	22.16 ± 2.33	21.69 ± 2.20	0.190
Corneal endothelial cell count (/mm)	2731.11 ± 312.30	2773.80 ± 250.64	0.448
Intraocular pressure (mmHg)	15.81 ± 2.81	15.12 ± 2.84	0.240
UDVA (logMAR)	0.76 ± 0.56	0.78 ± 0.61	0.786

OD, ocular dexter; OS, ocular sinister; IOL, intraocular lens; UDVA, uncorrected distance visual acuity.

### 3.2. Postoperative visual acuity

There was no significant difference in the best CDVA between the two groups 3 months after surgery (*p* > 0.05); the UDVA in the ZMT group was better than that in the ZXR group (*p* < 0.005), the UIVA in the ZXR group was better than that in the ZMT group (*p* < 0.001), and the UNVA in the ZMT group was better than that in the ZXR group (*p* < 0.001) (Table 2).

**Table 2:** Comparison of visual acuity and diopter 3 months after the operation.

Parameter	Mean ± SD		*p-*value
	ZXR	ZMT	
UDVA (logMAR)	0.13 ± 0.09	0.05 ± 0.07	0.001^**^
CDVA (logMAR)	0.01 ± 0.05	0.02 ± 0.05	0.813
UIVA (logMAR)	0.15 ± 0.09	0.30 ± 0.12	*p* < 0.001^***^
UNVA (logMAR)	0.35 ± 0.17	0.08 ± 0.08	*p* < 0.001^***^
IOL rotation (*D*)		2.50 ± 1.66	
Sphere (*D*)	−0.17 ± 0.50	0.03 ± 0.40	0.065
Cylinder (*D*)	−1.23 ± 0.31	−0.35 ± 0.15	<0.001^***^

IOL, intraocular lens; UDVA, uncorrected distance visual acuity; CDVA, corrected distance visual acuity; UIVA, uncorrected intermediate visual acuity; UNVA, correct near visual acuity (**p < 0.01, ***p < 0.001).

### 3.3. Defocus curve

**Figure 1:**
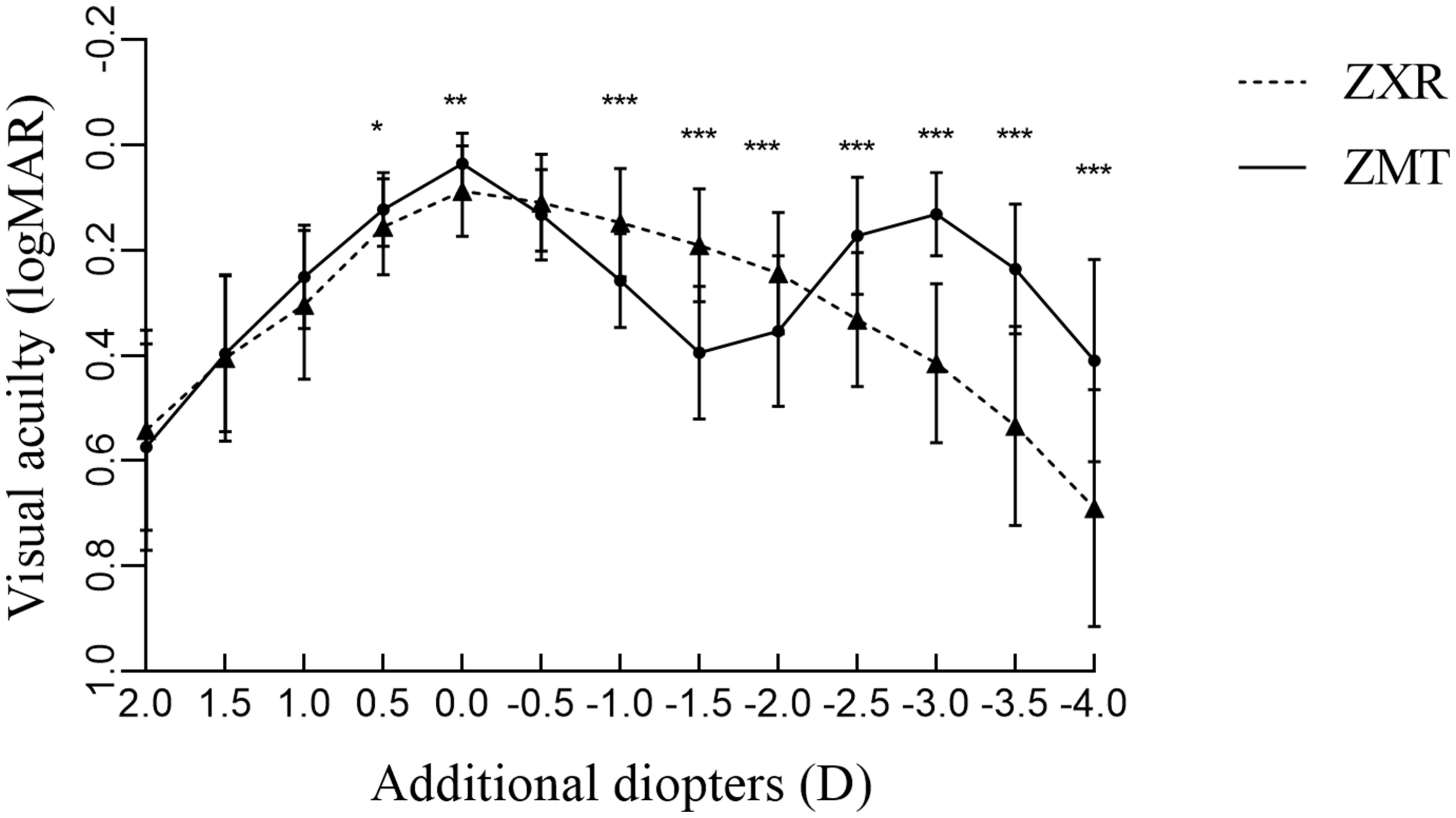
Comparison of defocus curves of patients 3 months after surgery (*p < 0.05, **p < 0.01, and ***p < 0.001).

In the ZXR group, visual acuity was in a plateau ranging from 0 to −1.5 D. The initial average visual acuity was >0.2logMAR which gradually decreased to −1.5–−4.0 D. The curve of the ZMT group showed a bimodal shape and an average visual acuity above 0.1logMAR. A visual acuity of 0 D (5 m distance) and −3.0 D (approximately 33 cm) were the best findings. The defocus curve of the ZXR group was better than that of ZMT at 0–−2.5 D and intersected at −2.5–−3.0 D. However, the visual acuity of the ZMT group was better than that of ZXR at −2.5-−4.0 D, as shown in Figure 1.

### 3.4. Refractive state

(i) *Corneal Astigmatism Diopter*: The absolute value difference of the corneal cylinders between the ZXR and ZMT groups pre- and post-operation were 0.18 ± 0.23 and 0.18 ± 0.12, respectively. There was no significant difference between the two groups (z = −1.373, *p* = 0.175 > 0.05).

(ii) *Residual Astigmatism Diopter*: There was no significant difference between the postoperative cylinder (−1.23 ± 0.31 D) and the pre-operative cylinder (−1.27 ± 0.14 D) in the ZXR group. The postoperative astigmatism in the ZMT group (−0.35 ± 0.15 D) was less than pre-operation astigmatism (−1.29 ± 0.14 D). The postoperative cylindrical diopter in the ZMT group was smaller than that in the ZXR group, and the difference was statistically significant (Table 2).

(iii) *Rotation Stability of the ZMT Group*: The rotation degree of ZMT IOL implanted 3 months after the operation was 2.50 ± 1.66 D (Table 2).

### 3.5. High order aberration and the MTF

**Figure 2:**
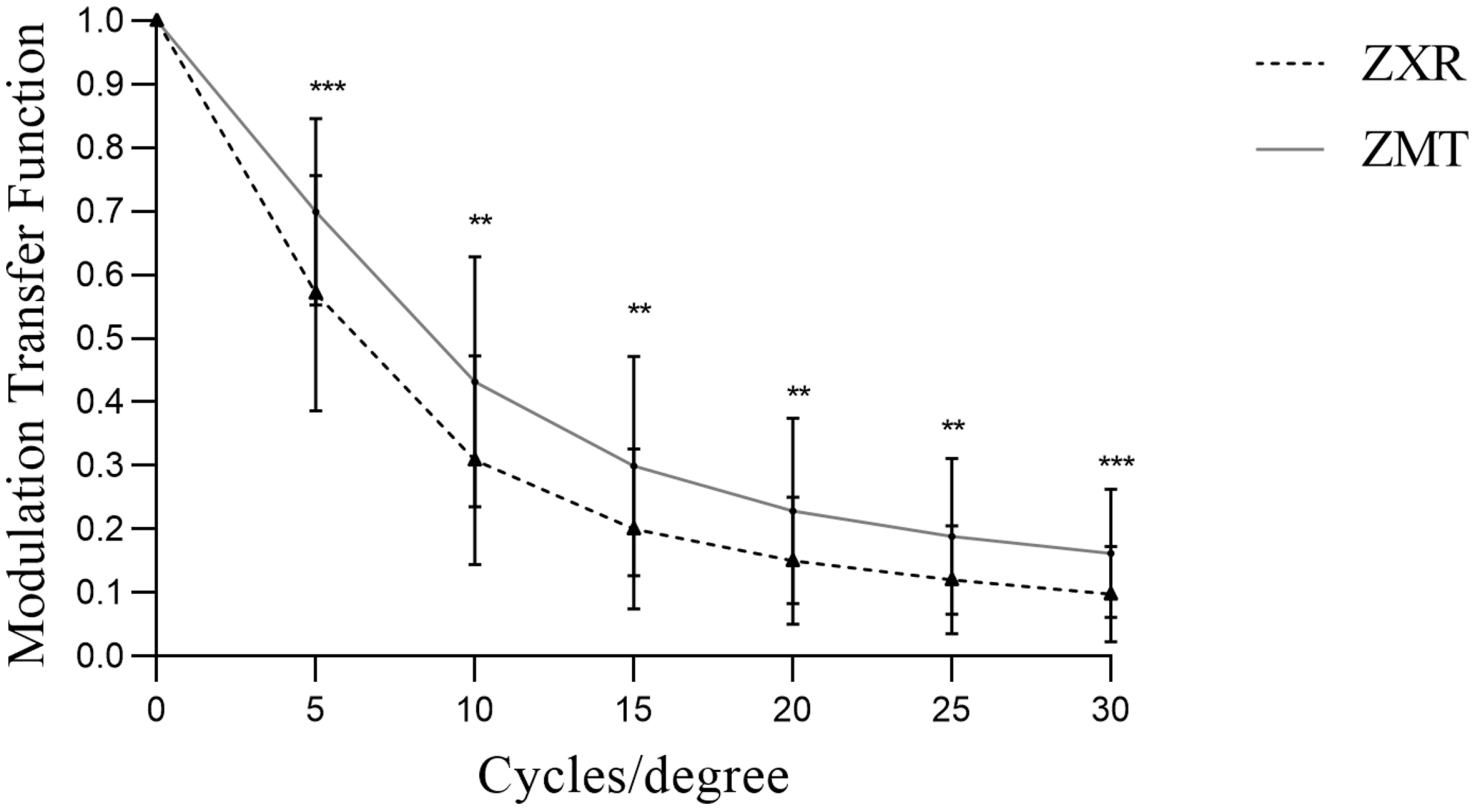
Comparison of the modulation transfer function (MTF) values under 3 mm pupil of patients 3 months after the operation (*p < 0.05, **p < 0.01, and ***p < 0.001).

The tHOA, coma, and trefoil in the ZMT group were lower than those in the ZXR group (*p* < 0.05), but there were no significant differences in SA (*p* > 0.05) (Table 3). There was no significant difference in the MTF of the cornea between the two groups; however, the mean MTF of the whole eye under a pupil size of 3 mm was significantly lower than that of the ZMT group (*p* < 0.05) (Table 3). There were also significant differences in the MTF values between the two groups at different spatial frequencies (*p* < 0.05), as shown in Figure 2.

**Table 3:** Comparison of aberrations and MTF values under 3 mm pupil at 3 months after the operation.

Parameter	Mean ± SD		*p-*value
	ZXR	ZMT	
tHO (μm)	0.19 ± 0.13	0.13 ± 0.06	0.014*
SA (μm)	−0.00 ± 0.04	0.00 ± 0.02	0.343
Coma (μm)	0.07 ± 0.05	0.05 ± 0.03	0.043*
Trefoil (μm)	0.11 ± 0.08	0.08 ± 0.06	0.030*
Corneal MTF	0.50 ± 0.16	0.50 ± 0.13	0.947
Mean MTF	0.32 ± 0.11	0.41 ± 0.13	0.001**

tHO, total high-order; SA, spherical aberration; MTF, modulation transfer function (*p <0.05, **p <0.01).

### 3.6. Questionnaire

**Figure 3:**
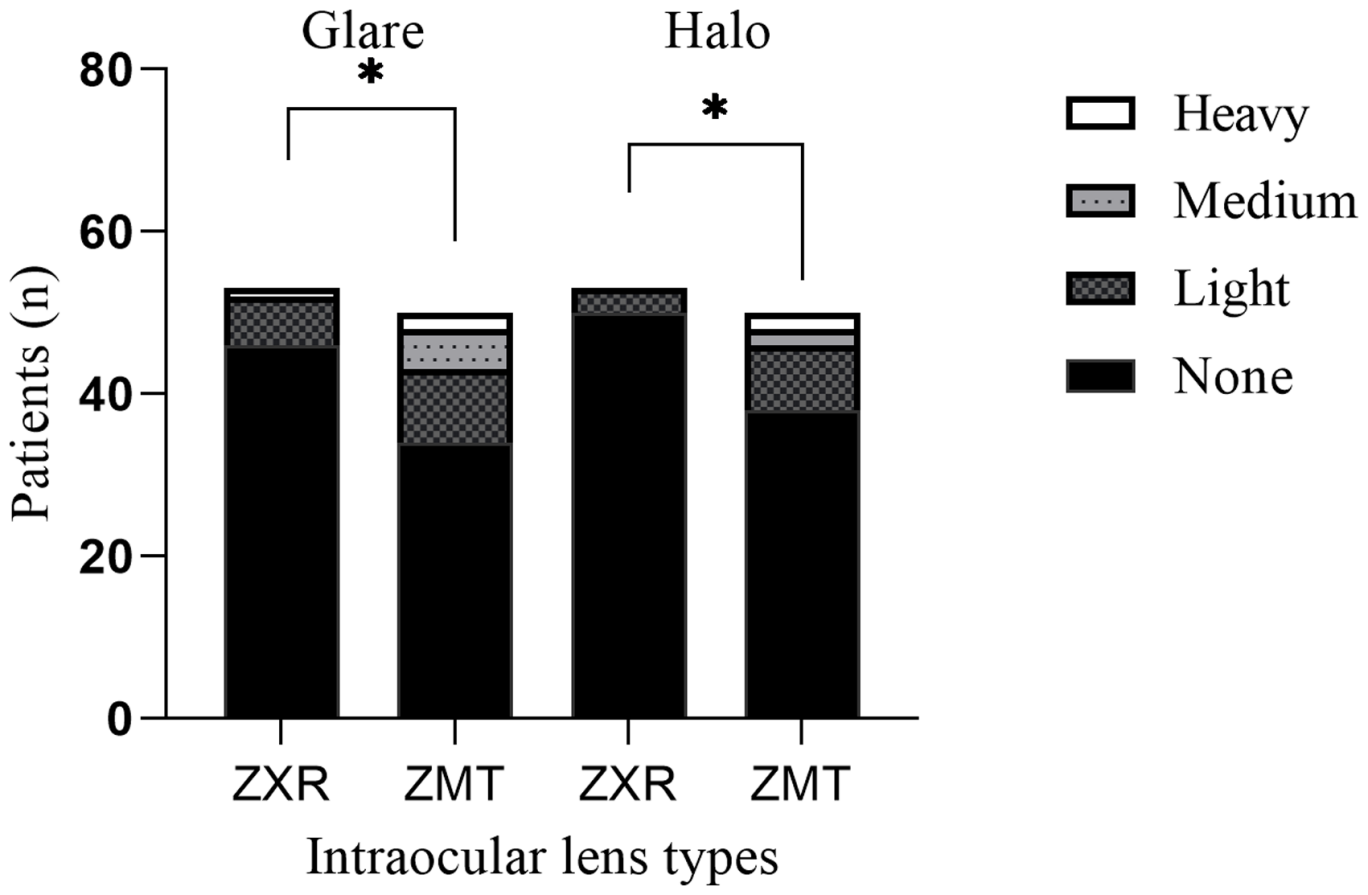
Comparison of adverse optical interference in postoperative patients in 3  months (*p < 0.05, **p < 0.01, and ***p < 0.001).

The postoperative VF14 score was higher in the ZMT group than in the ZXR group (Table 4). Comparing the subjective adverse optical interference between the two groups, the number of patients with glare and halo in the ZMT group was significantly higher than that in the ZXR group (*p* < 0.05), as shown in Figure 3. There was no significant difference in spectacle independence.

**Table 4:** Comparison of questionnaires and spectacle independence at 3 months after the operation.

Parameter	Mean ± SD		*p-*value
	ZXR	ZMT	
VF14	89.02 ± 4.46	91.57 ± 3.46	0.002**
Spectacles independence	52 (98.11%)	50 (100%)	
Glare			0.037*
None (*n*/%)	46 (86.8%)	34 (68.0%)	
Light (*n*/%)	6 (11.3%)	9 (18.0%)	
Medium (*n*/%)	0 (0%)	5 (10.0%)	
Heavy (*n*/%)	1 (1.9%)	2 (4.0%)	
Halo			0.025*
None (*n*/%)	50 (94.3%)	38 (76.0%)	
Light (*n*/%)	3 (5.7%)	8 (16.0%)	
Medium (*n*/%)	0 (0%)	2 (4.0%)	
Heavy (*n*/%)	0 (0%)	2 (4.0%)	

IOL, intraocular lens; VF14, visual function; QoV, quality of vision (*p <0.05, **p <0.01).

## 4. Discussion

In order to improve the postoperative visual function and quality of life for cataract patients, it is crucial to correct excessive astigmatism. Various methods, such as main corneal incision (PI), excimer laser *in situ* keratectomy (LASIK), astigmatic keratectomy (AK/FSAK), limbal release keratectomy (LRIS), femtosecond laser non-penetrating interlamellar astigmatism keratectomy (ISAK), and astigmatism correction intraocular lens implantation, can be employed (15–18). However, when taking into consideration the cost of surgery, complications, and the accuracy of astigmatism correction, toric intraocular lens implantation stands as a more suitable option for cataract patients. In this study, we provided a comparative analysis of Tecnis ZMT and Symphony ZXR00 IOLs to assess the visual quality of two different types of intraocular lens following cataract surgery with astigmatism. As far as we know, this is the first comparative analysis of its kind.

The uncorrected distance visual acuity in the ZMT group was found to be better than that in the ZXR group. The uncorrected astigmatism found in the ZXR group had a perceptible impact on the UDVA, whereas the ZMT group showed effective correction of astigmatism yielding good UDVA. The UIVA of the ZXR group was better, fully demonstrating the advantages of the EDoF IOLs extended visual range (19). Our findings revealed a naked near visual acuity (UNVA) of less than 0.2logMAR in the ZMT group, with the ZMT IOL near addition +4.0 D design enabling comfortable and clear near vision. Other studies have also observed comparable findings concerning the UNVA of ZMT (10, 11).

The defocus curve can be used to simulate the vision of the patient at different distances, and the accommodative range of the intraocular lens can be evaluated (20). Both lens provided good recovery of postoperative distant visual acuity. ZXR allowed for a more continuous distant and intermediate visual acuity from +0.5D to −2.0D, of a value above 0.2logMAR. The bimodal defocus curve also provided better near vision. The defocus curve shape is similar to that of Chang et al. (13, 21). Carones et al. found that the ZXR00 IOL has a higher tolerance for astigmatism than other types of bifocal and trifocal intraocular lens, which is related to the design of the ZXR00 IOL 1.6 mm large central apertures (22). Cylindrical lens of varying diopters were added in front of the patients’ eyes post-cataract implantation with ZXR00 IOL, and uncorrected distance vision was observed. Results demonstrated that postoperative residual astigmatism impacted distance vision (22).

High-order aberrations have a significant impact on the visual quality of patients, and MTF serves as a well-established standard for reflecting objective visual imaging. ZMT IOL effectively tackled the astigmatism, but residual astigmatism persisted after ZXR00 IOL surgery. We found that astigmatism may increase high-order aberrations (23, 24), mainly coma and trefoil (25), aligning with previous study findings. Additionally, the rotational stability design principle of ZMT IOL played a role in optimizing its objective visual quality. Ruiz-Alcocer et al. (26) previously highlighted that IOL rotation beyond 5D could impede overall visual quality. Based on our analysis, it is plausible to posit that the variations observed in objective visual quality indicators can be attributed to the combined effects of ZMT IOL correction for astigmatism and rotational stability.

During the postoperative follow-up, we found that the halo and glare phenomenon in the ZMT group was more serious than that in the ZXR group. As per our previous research, it has been observed that ZXR00 portrays a diminished occurrence of halos when compared to ZMB00, which is a diffractive bifocal IOL that shares similar design attributes with ZMT IOL (27). The ZXR00 IOL has a wide central optical zone (1.6 mm in diameter) and a large central step diameter, resulting in a reduced number of diffraction apertures and refraction of light. Additionally, ZXR00’s achromatic technology and low additional diopter incorporated in its echelette diffraction grating can reduce the occurrence of glare and halos while minimizing the loss of contrast sensitivity (28). The ZXR00 IOL also displays a light energy utilization rate of 92%, whereas bifocal IOLs employ a light-splitting design principle that limits the light allocated to each focus. Despite the potential for increased aberration with a larger pupil, the ZXR00 IOL’s large central ring design maintains excellent visual function with a pupil size of 4.5 mm (29). While postoperative glare can significantly impact visual cortex activation during the early stages of recovery, studies indicate that such disturbances typically dissipate over time (30, 31).

The VF14 score was higher in the ZMT group, which is presumably a result of the lens’s ability to correct astigmatism and provide better near vision correction for presbyopia in a single operation (14). Extensive research has shown that the ZMB00 IOL provides good near vision, and the addition of astigmatism correction with the ZMT IOL offers further benefits (32–35). Liu et al. (21) found higher VF-14 scores for the ZXR00 IOL group than the ZMB00 group, which differs from our findings. We speculate that the uncorrected astigmatism of ZXR00 caused lower scores in this study. Wolffsohn et al. (5) found that levels of uncorrected astigmatism as low as 1.00 D can significantly impact visual function and quality of life. In contrast, correction of astigmatism can effectively improve the quality of life of patients (36).

The limitations of our study are as follows: firstly, given the varying aberrations across different pupil sizes, it is advisable to undertake a broader visual quality analysis for larger pupils. Secondly, further examination on the astigmatism tolerances of the ZXR00 IOL lens can be done by grouping astigmatism degrees. Lastly as our study measured near visual acuity at a distance of 40 cm, we suggest that 33 cm, the habitual distance of Asian eyes, could be adopted as the distance of near visual acuity for future studies.

## 5. Conclusion

The ZMT IOL exhibited proficient near and distant vision, effectively correcting astigmatism, while the ZXR00 IOL provided an extended visual range and was found to be reasonably tolerant to astigmatism, primarily regarding its subjectively evaluated optical quality and range of vision. These findings offer essential information to guide refractive cataract surgery for clinicians and improve the future of eye health.

## Data availability statement

The original contributions presented in the study are included in the article/supplementary material, further inquiries can be directed to the corresponding author.

## Ethics statement

Written informed consent was obtained from the individual(s) for the publication of any potentially identifiable images or data included in this article.

## Author contributions

ZL: conceptualization, methodology, data curation, formal analysis, and writing—original draft. RG: methodology and data curation. XH, XY, ZW, and YL: data curation. HZ: funding acquisition, project administration, resource, and writing—review and editing. All authors contributed to the article and approved the submitted version.

## Funding

This paper was funded by the International Science and Technology Cooperation Project of the Department of Science and Technology of Jilin Province (20200801026GH) and Natural Science Foundation of Jilin Province (YDZJ202301ZYTS038).

## Conflict of interest

The authors declare that the research was conducted in the absence of any commercial or financial relationships that could be construed as a potential conflict of interest.

## Publisher’s note

All claims expressed in this article are solely those of the authors and do not necessarily represent those of their affiliated organizations, or those of the publisher, the editors and the reviewers. Any product that may be evaluated in this article, or claim that may be made by its manufacturer, is not guaranteed or endorsed by the publisher.

## Footnotes

^1^ https://www.Tecnistoriccalc.com
